# Mapping of the adherence to the planetary health diet in 11 European countries: comparison of different diet quality indices as a result of the PLAN’EAT project

**DOI:** 10.3389/fnut.2025.1645824

**Published:** 2025-09-17

**Authors:** Federica Grant, Vittoria Aureli, Jacopo Niccolò Di Veroli, Laura Rossi

**Affiliations:** ^1^CREA - Council for Agricultural Research and Economics, Research Centre for Food and Nutrition, Rome, Italy; ^2^Department of Agricultural, Environmental and Food Sciences (DiAAA), University of Molise, Campobasso, Italy; ^3^Department of Agricultural, Forestry and Food Sciences (DISAFA), University of Turin, Grugliasco, Italy; ^4^Departmental Faculty of Sciences and Technologies for Sustainable Development and One Health, University Campus Bio-Medico of Rome, Rome, Italy; ^5^Department of Food Safety, Nutrition and Veterinary Public Health, National Institute of Health, Rome, Italy

**Keywords:** dietary patterns, diet indices, health, sustainability, PLAN’EAT project, European countries, cluster analysis

## Abstract

**Introduction:**

The Planetary Health Diet (PHD) is a nutritional approach integrating public health and environmental sustainability aspects. This study, conducted within the European PLAN’EAT project, aimed to assess adherence to PHD in 11 European countries.

**Methods:**

Three dietary quality indices were used: the EAT-Lancet index; the original WISH; and a newly developed version, WISH 2.0. This last index incorporates two additional food categories: processed meat and alcoholic beverages. The inclusion of these categories was driven by their recognized public health and environmental relevance. The food consumption data was retrieved from the EFSA Comprehensive European Food Consumption Database. Scores were calculated and normalized to carry out descriptive and comparative analyses. Cluster analyses were also performed to examine dietary pattern differences by country and gender.

**Results:**

Low adherence to PHD was observed across all countries. However, Southern European countries such as Italy, Greece, and Spain showed comparatively higher adherence, particularly among women. Cluster analyses, based on EAT-Lancet and WISH 2.0 scores, highlighted regional and gender patterns. These findings emphasize the cultural specificity of dietary behaviors. The two indices have different discriminating capacities. From the EAT-Lancet index, higher average normalized scores were obtained. WISH 2.0 could distinguish between different dietary patterns and was better aligned with actual food consumption data, demonstrating an enhanced capacity to better detect national dietary patterns more accurately.

**Discussion:**

These results underscore the potential of WISH 2.0 as a comprehensive and practical instrument for mapping and monitoring dietary quality in Europe. Additionally, the findings indicate that adapting global dietary guidelines to local contexts may be essential to improve population-level adherence and policy relevance.

## Graphical abstract

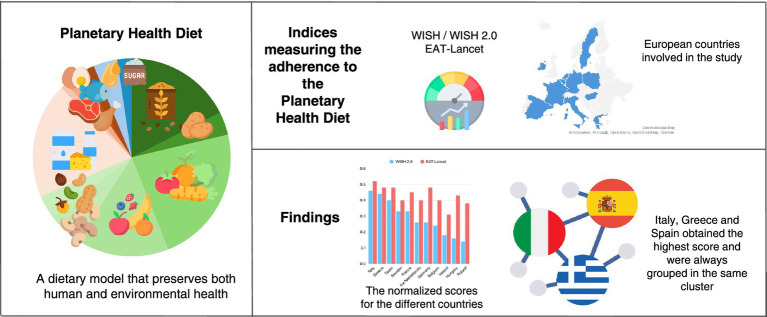

## Highlights

- The 11 European countries studied showed low adherence to the Planetary Health Diet, indicating a substantial gap between current dietary patterns and the nutritional recommendations aimed to promote human health and environmental sustainability.
- Southern European countries, particularly Italy, Greece, and Spain, showed the highest adherence to the Planetary Health Diet, highlighting its similarities with the Mediterranean diet. Both dietary patterns share key characteristics, including a high consumption of plant-based foods and limited intake of animal products.
- Gender-based analyses revealed that women exhibited dietary behavior that was more in line with the Planetary Health Diet recommendations than men. This finding is consistent with existing literature that identifies women as being more likely to engage in health-conscious dietary behaviors.
- The newly developed WISH 2.0 offered greater discriminatory capacity, providing a more accurate reflection of ongoing food consumption patterns. Therefore, WISH 2.0 can be considered a more effective tool for monitoring the dietary quality of European populations.

## 1 Introduction

Food systems have gained a pivotal role in the ongoing climate crisis, highlighting the need to transform food production and consumption to achieve Sustainable Development Goals (SDGs) by 2030. The agri-food sector is responsible for up to 30% of anthropogenic Green House Gas (GHG) emissions, uses 70% of freshwater resources, contributes to 78% of water eutrophication, and occupies half of the inhabitable land (1). In this framework, the livestock sector (especially ruminants) is the primary contributor to GHG emissions (2, 3). In 2020, agrifood systems contributed over 30% of the total GHG emissions in the European Union (EU) (4). Food systems are also facing great challenges due to the high demand driven by a growing global population. In 2021, over 3.1 billion people (42% of the world’s population) could not afford a healthy diet, representing an increase of 134 million since 2019 (5). High consumption levels of animal products, processed foods, saturated fats, sugar, and salt and low intake of plant-based products represent the most common dietary risks of the current diets. According to the Global Burden of Disease Study, in 2019, dietary risks accounted for 7.9 million deaths and 187.7 million reported disability-adjusted life-years (DALYs) (6). These environmental and dietary challenges are central to current European public health and sustainability agendas. Different EU programs, such as the European Green Deal (7) and, particularly, the Farm to Fork Strategy (8), highlight the urgent need to shift toward healthier and more sustainable food systems. In addition to that, an emphasis on the importance of tools that can monitor dietary patterns and inform policy decisions was highlighted. In this context, the connection between environmental and human health leads to a reconsideration of food policies advocating for an integrated approach that address both aspects. As outlined by the Food and Agriculture Organization (FAO) and the World Health Organization (WHO) in 2019, a sustainable and healthy diet must address health, environmental, and socio-cultural factors (9).

Building on this foundation, the EAT-Lancet Commission proposed the Planetary Health Diet (PHD), a predominantly plant-based dietary model recognized for its health benefits. This dietary pattern is designed to have a minimal environmental footprint, where meat and dairy products represent a small portion of the proposed dietary pattern (10).

Several indicators have been developed to assess the dietary quality of the population. As highlighted by Harrison MR et al. (11), effective indicators are essential for promoting sustainable and healthy diets based on evidence. They also play a key role in tracking food consumption progress at the national and local levels. As a modern approach, diet quality indices have evolved to incorporate both health and sustainability aspects. Examples include the Sustainable-Healthy-Diet (SHED) Index, which is based on adherence to the Mediterranean Diet (MD) (12), the Healthy and Sustainable Diet Index (SNRF), which incorporates recommendations from the Australian Dietary Guidelines (13), and the Sustainable Nutrient Rich Foods (SNRF) index, which is based on the health-related nutritional characteristics and greenhouse gas emissions of foods (14).

The publication of the EAT-Lancet recommendations has further stimulated the development of other indicators that use the PHD as a reference. These include the Planetary Health Diet Index (PHDI) (15), EAT-Lancet index (16), the World Index for Sustainability and Health (WISH) score (17), the Healthy Reference Diet (HRD) index (18), the EAT-Lancet Diet Index (19), the EAT-Lancet diet score with minimum intake values (20), and the EAT-Lancet Diet score (ELD-I) (21). However, the methodologies used to develop and validate these indicators vary. A comparative analysis highlighting their similarities and differences could offer guidance in the selection of indices to apply while considering their weaknesses and strengths. In this regard, it is worth noting the study of Stubbendorff A et al. (22). Different scores of three cohorts were examined to assess how consistently they measure adherence to the PHD recommendations. While no index was clearly superior, the results indicated that the EAT-Lancet index and, to a lesser extent, the Healthy Reference Diet (HRD) were the most effective tools for assessing adherence to the PHD.

In the context of the European Horizon PLAN’EAT Project (23), which aimed to provide data and recommendations to transform the food system toward healthier and more sustainable dietary behavior, an adapted version of the WISH score (17) was created and applied. This updated version, named WISH 2.0, expanded the original WISH score by including two additional food categories: processed meat and alcoholic beverages. These categories were added due to their recognized relevance for public health and sustainability (24). WISH 2.0 was used to map the food quality level of the 11 countries participating in the PLAN’EAT project (25). The WISH, WISH 2.0, and EAT-Lancet index all share the common characteristic of being based on food group consumption rather than nutrients. This makes them practical, user-friendly tools that well align with recommendations provided in food-based dietary guidelines.

Based on the outcome of the PLAN’EAT project, this study compared WISH and WISH 2.0, which are both calculated using a continuous scoring system, with the EAT-Lancet index, which applies an ordinal scoring system. The Healthy Reference Diet (HRD) index, identified by Stubbendorff et al. (22) as high-performing, was excluded from this study due to its structural similarity to WISH and WISH 2.0 and because its continuous scoring system offered limited added value for comparison.

The hypotheses underlying this study are the followings (i) the use of different indices to assess adherence to the Planetary Health Diet can reveal cultural eating habits differences across 11 European countries and between genders; and (ii) countries with dietary patterns traditionally aligned with plant-based diets (e.g., Mediterranean countries) are expected to achieve higher scores compared to countries with more animal-based dietary patterns.

Against this background, the purpose of this study is threefold: (1) to present the development of the WISH 2.0 score, which represents an evolution of WISH. This new version incorporates two additional food categories (processed meat and alcoholic beverages) which are relevant both for human health and for environmental impact; (2) to compare the newly developed WISH 2.0 with the EAT-Lancet index, which, according to Stubbendorff A et al. (22), outperformed other indicators. The comparison aims to highlight differences and similarities between the two indices and to assess which is more effective in capturing variations in dietary patterns across the 11 countries analyzed in the PLAN’EAT project; and (3) to assess how closely the 11 countries of this study adhere to the PHD recommendations, identifying dietary patterns observed both across countries and between genders.

## 2 Materials and methods

### 2.1 Study design

In the first phase of this study, a literature analysis of diet quality indicators was carried out. PubMed, Scopus, and Google Scholar databases were consulted using the following keywords: health OR healthy AND index; sustainable AND index; health OR healthy AND sustainable AND index; health OR healthy AND sustainable AND diet AND index. Considering the aim of this study, the selection process identified diet quality indices that accounted for both health and sustainability aspects, were based on food groups, and were applicable to existing food consumption datasets. This led to the selection of the EAT-Lancet index developed by Stubbendorf et al. (16), WISH developed by Trijsburg et al. (17), and WISH 2.0 developed in the framework of the PLAN’EAT project (26).

### 2.2 Food consumption data selection

The EFSA Comprehensive European Food Consumption Database, which is publicly available, was used to apply the different indices (27). This dataset is designed to assess the nutrient intake among EU consumers and evaluate the potential risks of consumer exposure to hazards. National food consumption data were collected using a homogeneous and standardized methodology (the EU menu methodology) (28). The most recent survey for each country was used to calculate the indices, providing food consumption data expressed in grams per day by gender, based on the mean individual consumption of the adult population (18–64 years). Summary statistics of food consumption data were analyzed according to the sixth level of the ‘Exposure Hierarchy’ in the FoodEx2 food classification and description system (27). The analysis covered the 11 European countries participating in the PLAN’EAT project, namely Belgium, France, Germany, Greece, Hungary, Ireland, Italy, Poland, Spain, Sweden, and the Netherlands.

### 2.3 Food category selection and indices’ development criteria

The diet quality indices used in this study—the EAT-Lancet index, WISH, and WISH 2.0—were designed to measure adherence to the PHD recommendations. The HRD index, although identified as a high-performing tool in previous comparative studies, was not included in this analysis due to its close conceptual and structural similarity to the WISH and WISH 2.0. To maintain a comparison framework and avoid redundancy, indices with greater conceptual diversity were prioritized. The food categories from the EFSA database associated with each index component are detailed in Supplementary Table S1. The inclusion of food categories was determined based on the development criteria of each index. Although the EAT-Lancet index, WISH, and WISH 2.0 share a common framework, there are differences between them. For instance, the EAT-Lancet index does not include ‘saturated oils’, whereas WISH and WISH 2.0 do not account for ‘potatoes’. Additionally, the EAT-Lancet index distinguishes between ‘beef and lamb’ and ‘pork’ as separate groups within the red meat category, whereas WISH and WISH 2.0 classify ‘red meat’ as a single category. Aligning the EFSA food categories with the index components required adjustments. For instance, a selection procedure was applied to determine which ‘processed meat’ categories should have been included in the EAT-Lancet index, since this index differentiates between meat sources based on the type of animal (e.g., ‘beef and lamb’ or ‘pork’). As EFSA food categories do not always correspond to a single type of meat, the predominant meat variety in a given food item was considered. Sausages, for example, which are commonly made with pork, were therefore assigned to the ‘pork’ food group. Furthermore, to quantify ‘added sugars’ intake, the food groups that contribute most to added sugar consumption at the European level were included, as identified by EFSA (29). The added sugar intake from these selected categories was calculated based on their proportion of total and free sugars (29).

Regarding the indices, the reference intake values from the PHD recommendations were used for food consumption scores, except in the case of whole-grain cereals in WISH and WISH 2.0. For this category, the recommendations from the Global Burden of Disease study (30) were applied, specifying an intake of 125 g/day (within a range of 100 and 150 g). An ordinal scoring system was used for the EAT-Lancet index, while a continuous scoring system was applied for WISH and WISH 2.0 (Supplementary Tables S2, S3). In the EAT-Lancet index, for each food category, a score from 0 to 3 was assigned, where 0 indicates a low adherence to the PHD recommendations and 3 represents high adherence. Intermediate scores between 2 and 1 were assigned based on the minimum and maximum levels within the recommended intake range. The proportional scoring system used for WISH and WISH 2.0 can be found in details within the study of Trijsburg et al. (17) and reported below. For each category, a score between 0 and 10 was assigned, where 0 indicates no adherence to the recommended intake and 10 represents complete adherence. Scores between 0 and 10 were calculated using formulas that differentiate between food categories that have positive or negative impact on human health and/or on the environment.

For whole grains, vegetables, fruit, dairy, fish, legumes, nuts, and unsaturated fats, the following formula was applied:


$$\frac{10*(\text{reported intake}-\text{lower recommended intake})}{\left(\text{recommended intake}-\text{lower recommended intake}\right)}$$

For red meat, processed meat, chicken and other poultry, and eggs, the following formula was applied:


$$\frac{10*(\begin{array}{l} \text{upper recommended intake}\\ -\text{recommended intake} \end{array})-(\begin{array}{l} \text{reported intake}\\ -\text{recommended intake} \end{array})}{\left(\begin{array}{l} \text{upper recommended intake}\\ -\text{recommended intake} \end{array}\right)}$$

For saturated oils and added sugars, cut-off points for intake were set at11.8 and 31 grams per day, respectively. Scores were assigned as bivariate components, equal to 0 if the consumption was below the cut-off point and equal to 10 for consumption above the cut-off point. As previously mentioned, WISH was revised to create WISH 2.0 (Supplementary Table S3), incorporating two additional food categories, ‘processed meat’ and ‘alcoholic beverages’, given their significant public health and environmental implications (31–34). To apply these changes, ‘processed meat’ was separated from the ‘red meat’ category, where it was originally grouped in both the EAT-Lancet index and WISH. Additionally, a new category was introduced: ‘alcoholic beverages’. The scoring calculation for these newly added food groups was designed to reflect their distinct dietary recommendations and public health impact. For the ‘processed meat’ category, the recommended intake established by the Global Burden of Disease study (2–4 g/day) (6, 28) was applied. Accordingly, a score of 10 points was assigned for consumption below 2 grams per day, and 0 points were assigned for consumption exceeding 4 grams per day. Scores between 0 and 10 were calculated using the same formula as that applied to the ‘red meat’ category in the original scoring system. For ‘alcoholic beverages’, the World Cancer Research Fund recommends avoiding this category completely as the best approach to reducing the risk of developing cancer (35). Therefore, a binary scoring system was used, where 10 points were assigned for no alcohol consumption, and 0 points were given for any intake above 0 grams per day. This approach was consistent with that used for the ‘saturated oils’ and ‘added sugars’ categories in the original scoring system.

### 2.4 Data analysis

A descriptive analysis was carried out to examine the dietary patterns in each country and to compare the WISH, WISH 2.0, and EAT-Lancet index. To account for differences in the scales of the two indices, a normalization procedure was applied. Specifically, the minimum-maximum normalization method was used to ensure that all scores fell within the 0–1 range. The indices were compared considering the minimum and maximum theoretical values. The following formula was used:


$$x_{i}^{\prime }=\frac{x_{i}-\min(x)}{\max(x)-\min(x)}$$

The EAT-Lancet index and WISH 2.0 were examined using cluster analysis. Specifically, an aggregate hierarchical clustering analysis was performed based on the points assigned to each food category in both indices, as well as the quantities of food consumed (in grams).

Gower’s distance metric and a generalized version of Ward’s linkage method were used for the hierarchical clustering analyses (34).

Grower’s distance between unit i and unit j was defined as follows:


$$d(i,j)=\frac{\sum_{k=1}^{v}\delta_{\mathrm{ijk}}\;d_{\mathrm{ijk}}}{\sum_{k=1}^{v}\delta_{\mathrm{ijk}}}$$

Where v represents the number of variables; $\delta_{\mathrm{ijk}}$ is a binary indicator variable, equal to 1 if observations 𝑖 and 𝑗 are comparable for variable *k*, and 0 otherwise; and $d_{\mathrm{ijk}}$ denotes the dissimilarity between observations 𝑖 and 𝑗 for variable *k*, which is defined as follows:


$$d_{\mathrm{ijk}}=\{\begin{array}{c} \text{if}\;k\;\text{is numeric}\to d_{\mathrm{ijk}}=\frac{|k_{i}-k_{j}|}{\text{Range}(k)}\;\\ \text{if}\;k\;\text{is categorical}\{\begin{array}{c} \mathrm{se}\;k_{i}=k_{j}\to d_{\mathrm{ijk}}=0\\ \mathrm{se}\;k_{i}\neq k_{j}\to d_{\mathrm{ijk}}=1 \end{array} \end{array}$$

Although Ward’s original method minimizes the increase in total within-cluster variance using squared Euclidean distances, a generalized formulation allows its application to non-Euclidean distances such as Gower’s distance. In this generalized approach, the objective function is reformulated so that the algorithm minimizes the total within-cluster increase in the least absolute deviation, rather than variance, at each agglomeration step, thus preserving the fundamental principle of Ward’s method (36). To determine the optimal cut point for the dendrogram, both the scree plot of cluster merging heights and the graph of average silhouette widths were examined.

The silhouette width for each *i* unit was calculated using the following formula:


$$S(i)=\frac{b(i)-a(i)}{\max(\;b(i),a(i)\;)}$$

where a(i) represents the average distance between point 𝑖 and all other points within the same cluster (excluding 𝑖), while b(i) represents the average distance between point 𝑖 and all points in the nearest neighboring cluster. Consequently, S(i) ranges between −1 and 1.

The overall silhouette score is computed as an average of the silhouette values for all units.

## 3 Results

### 3.1 Overview of the country’s scores

As shown in Table 1, the average total score for the 11 analyzed countries using the EAT-Lancet index was 18.1, lower for men (16.9) and higher for women (18.6). The mean total score of observations using both WISH and WISH 2.0 scores was 43.7. Men had a lower average score (39.8) compared to women (47.1). No differences were observed in the overall WISH and WISH 2.0 scores, as the two food categories added to WISH 2.0 both scored 0 in all countries. Supplementary Tables S4, S5 detailed the scores assigned to each country for each food category.

**Table 1:** The final scores of the three indices applied to the 11 European countries analyzed, presented for the entire population and differentiated by gender.

Country	Total			Female			Male		
	WISH 2.0	WISH	EAT	WISH 2.0	WISH	EAT	WISH 2.0	WISH	EAT
Italy	**68.6**	**68.6**	**22**	**69.8**	**69.8**	**23**	**56.9**	**56.9**	**20**
Greece	66.6	66.6	20	66.5	66.5	21	56.5	56.5	**20**
Spain	59.8	59.8	20	63.0	63.0	20	56.7	56.7	19
Sweden	50.0	50.0	17	52.0	52.0	17	39.3	39.3	14
France	49.3	49.3	19	50.4	50.4	19	47.7	47.7	17
the Netherlands	39.2	39.2	17	40.3	40.3	18	36.5	36.5	16
Germany	39.1	39.1	20	41.6	41.6	19	38.0	38.0	**20**
Belgium	36.1	36.1	17	47.9	47.9	18	33.2	33.2	17
Ireland	27.2	27.2	**13**	29.7	29.7	**15**	26.3	26.3	**13**
Hungary	23.7	23.7	18	36.6	36.6	20	26.0	26.0	17
Poland	**21.1**	**21.1**	16	**20.8**	**20.8**	**15**	**20.7**	**20.7**	**13**
MEAN	**43.7**	**43.7**	**18.1**	**47.1**	**47.1**	**18.6**	**39.8**	**39.8**	**16.9**

The color gradient highlights the score ranking, from green (the highest score) to red (the lowest score). The bold values indicate the maximum and minimum score obtained for each country for all indices (WISH 2.0, WISH, EAT) together with the mean value score obtained by each country.

### 3.2 Index comparison after theoretical minimum and maximum normalization

**Figure 1:**
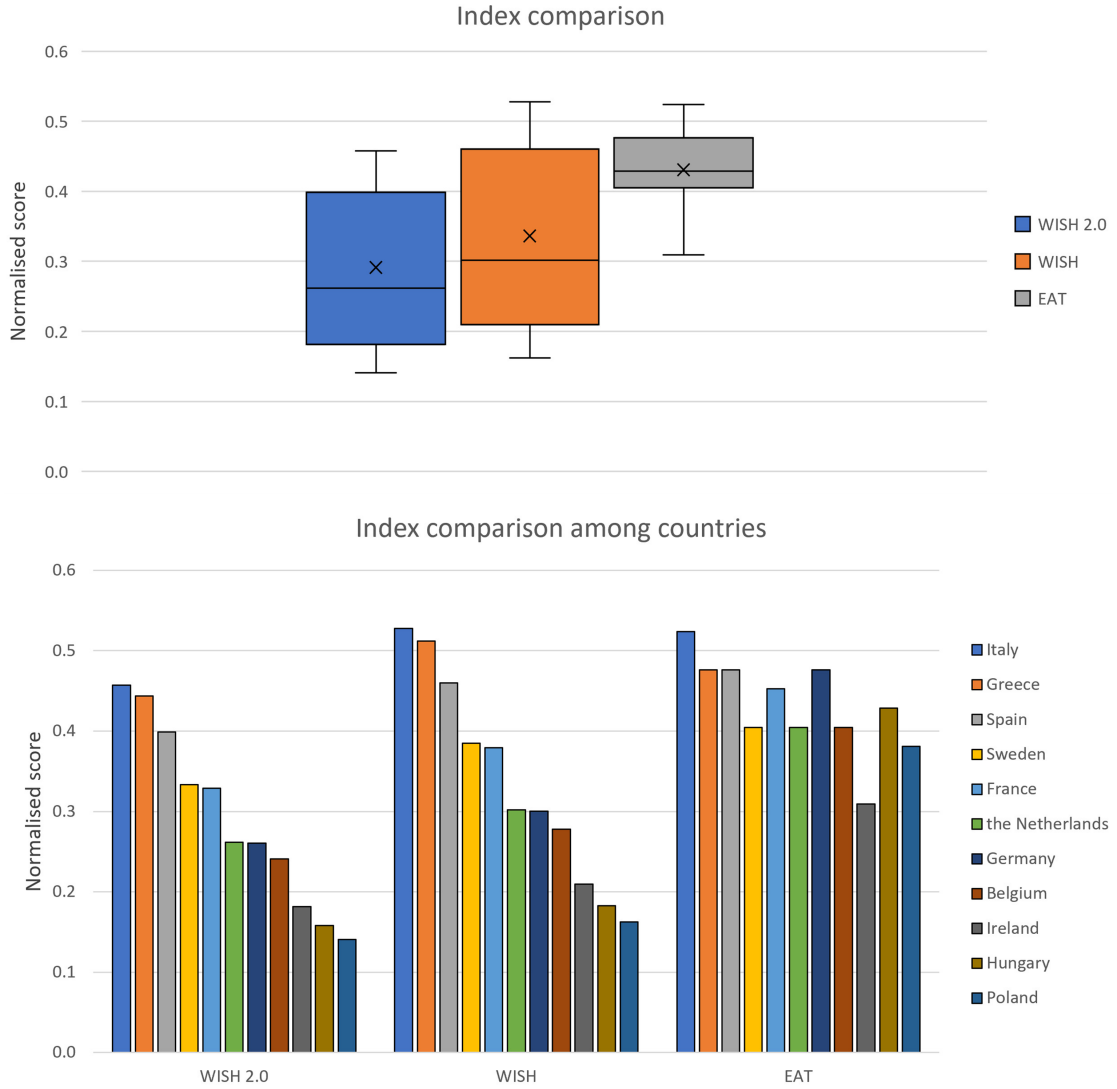
Indices comparison. Boxplot and bar plot showing a comparison of the indices normalized to the theoretical minimum and maximum scores for the whole population. EAT-Lancet index: minimum 0, maximum 42; WISH 2.0: minimum 0, maximum 150; WISH: minimum 0; maximum 130.

As shown in Panel A of Figure 1, after normalizing the theoretical minimum and maximum scores of each index (EAT-Lancet index: minimum 0, maximum 42; WISH 2.0: minimum 0, maximum 150; WISH: minimum 0; maximum 130), the EAT-Lancet index (the grey box) had a higher average value (0.43), whereas the values of WISH (yellow box) and WISH2.0 (blue box) were lower (0.34 and 0.29, respectively). Additionally, the EAT-Lancet scores resulted in lower variability (SD: 0.059) than WISH 2.0 (SD: 0.111) and WISH (SD: 0.128).

Comparing the normalized scores of the three indices across the 11 analyzed countries (panel B of Figure 1), Italy, Greece, and Spain consistently achieved the highest scores. However, Ireland obtained the lowest EAT-Lancet index score and Poland achieved the lowest WISH2.0 and WISH score. When the normalized results of the three indices were compared, the results showed that most countries obtained a higher EAT-Lancet index score. The only exceptions were Italy and Greece, where the WISH and WISH 2.0 scores were higher. Italy had similar scores for the EAT-Lancet and WISH (0.52 and 0.53 respectively) and a lower score for WISH 2.0 (0.46). Greece had a slightly higher score for WISH (0.51) compared to EAT-Lancet (0.48) and a lower score for WISH 2.0 (0.44). However, the difference between WISH 2.0 and the WISH score was due to the addition of the two food categories scoring zero, which reduced the final normalized WISH 2.0 value.

### 3.3 Cluster analyses

Five cluster analyses were carried out on the points assigned to each food category included in the WISH 2.0 and the EAT-Lancet indices, together with the grams of foods consumed in the 11 PLAN’EAT European countries. The WISH was not included in these analyses, as the two distinguishing items of both WISH 2.0 and WISH scored zero in all countries. Analyses were carried out for both the WISH 2.0 and EAT-Lancet indices, using the scores for the total population and differentiated by gender. To interpret the results of the cluster analyses in the light of European regional characteristics, the division into four macro-regions, as defined by the Global Nutrition Report (37), was considered. The 11 PLAN’EAT countries were therefore located as follows: North was Sweden and Ireland; East was Hungary and Poland; West was Belgium, France, Germany, and the Netherlands; and South was Italy, Spain, and Greece.

#### 3.3.1 Cluster analysis based on the WISH 2.0 score

##### 3.3.1.1 The structure of clusters and their alignment with index ranking

**Figure 2:**
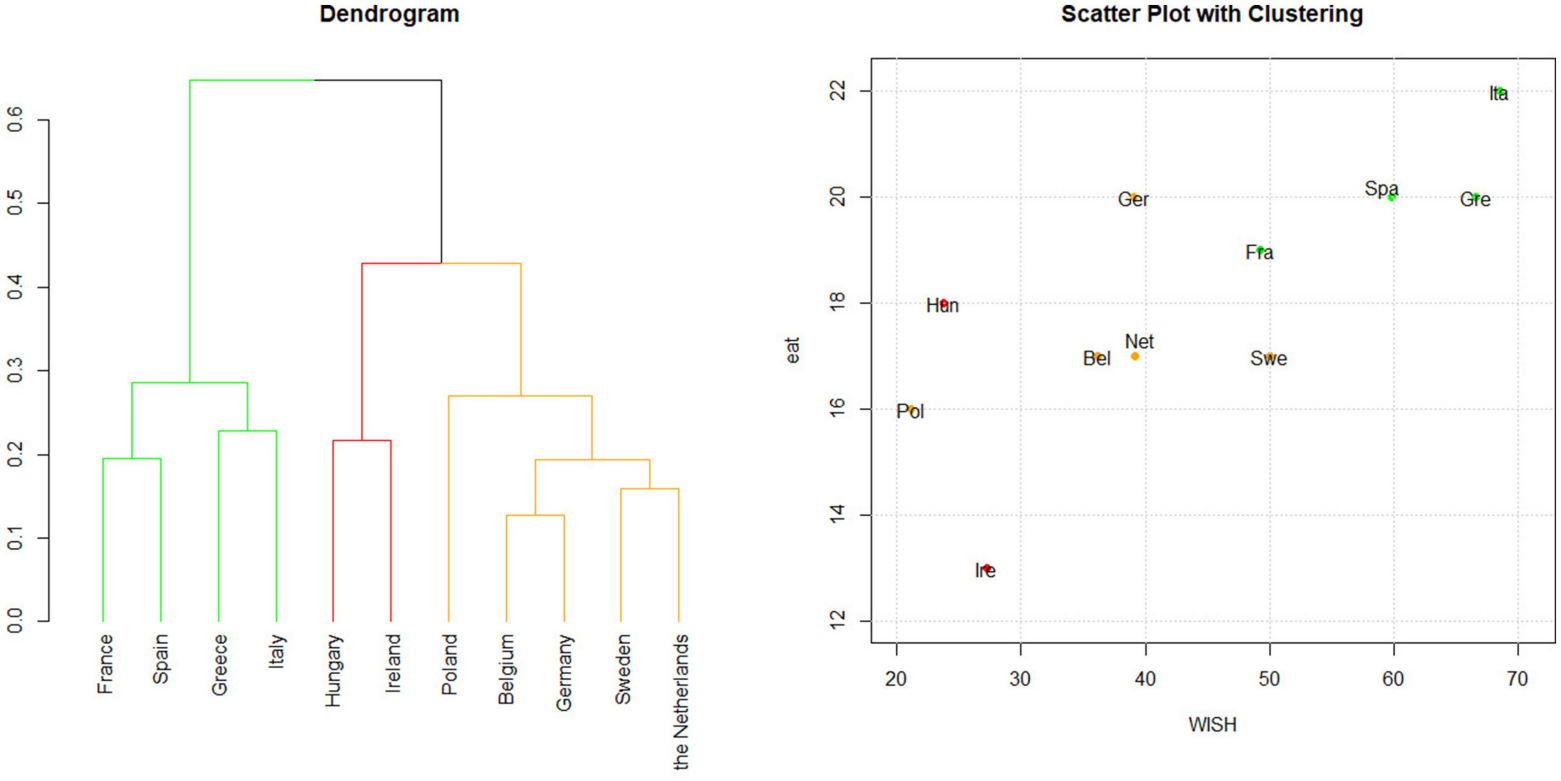
Hierarchical clustering based on WISH 2.0. Cluster analysis was applied to the points assigned to each food category using the WISH 2.0 index system. Dendrogram and scatter plot showing the results of the cluster analysis conducted in the 11 PLAN’EAT countries. The dendrogram shows how the countries are grouped according to clustering, while the scatter plot shows WISH 2.0 and EAT-Lancet indices’ scores for each country. The plots are colored according to the cluster groups.

Cluster analysis was applied to the points assigned to each category of food consumed according to the WISH 2.0 system (Figure 2). The three clusters obtained (green, yellow, and red) largely reflected the ranking achieved by the WISH 2.0 score of the whole population (Supplementary Table S6).

Countries in the *green cluster*, Italy, Greece, Spain, and France, obtained the highest WISH 2.0 total scores, indicating strong adherence to the PHD recommendations. The *yellow cluster,* which included Belgium, Germany, the Netherlands, Sweden, and Poland, comprised countries with an intermediate score. However, Poland showed lower adherence than both Ireland and Hungary, which are part of the *red cluster* representing the countries with the lowest scores.

In addition to the discrepancy between Poland’s ranking and its cluster assignment, a similar result was observed for Sweden and France. Despite having comparable WISH 2.0 scores (50 and 49, respectively), these countries were placed in different clusters: Sweden was grouped in the intermediate-score cluster (*yellow*), while France was included in the high-score cluster *(green)*. This divergence may reflect differences in dietary habits. Sweden’s eating patterns were more aligned with those of other Western European countries, whereas France’s eating patterns partially aligned with those typical of Southern European countries.

Moreover, analyzing the silhouette values enabled this output to be fine-tuned, considering that France’s silhouette value (0.07) placed this country between green and red clusters (Supplementary Table S6).

##### 3.3.1.2 Regional patterns

With respect to macro-regions, the green cluster included countries of the Mediterranean area, incorporating Southern European countries and France; in contrast, the yellow and red clusters exhibited a more heterogeneous inclusion of countries from the remaining macro-regions.

#### 3.3.2 Cluster analysis based on the EAT-Lancet index score

##### 3.3.2.1 The structure of clusters and their alignment with index ranking

**Figure 3:**
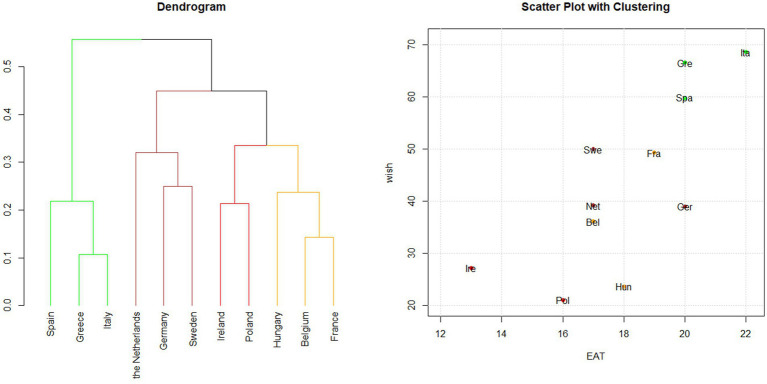
Hierarchical clustering based on the EAT-Lancet index. Cluster analysis was applied to the points assigned to each food category using the EAT-Lancet index system. Dendrogram and scatter plot showing the results of the cluster analysis conducted in the 11 PLAN’EAT countries. The dendrogram shows how the countries are grouped according to clustering, while the scatter plot shows EAT-Lancet and WISH 2.0 indices’ scores for each country. The plots are colored according to the cluster groups.

Cluster analysis was performed on the points assigned to each category of food consumed based on the EAT-Lancet index system (Figure 3). The four clusters were partially aligned with the overall EAT-Lancet index ranking (Supplementary Table S7) but they differed from the clustering obtained using WISH 2.0. The *green* cluster included only Southern European Countries—Italy, Greece, and Spain—which achieved the highest scores, indicating strong adherence to the PHD recommendations. In this analysis, France was grouped within the *yellow* cluster, alongside Belgium and Hungary, suggesting moderate adherence. The *brown* cluster (the Netherlands, Germany, and Sweden) also showed moderate adherence. Conversely, Ireland and Poland, within the *red* cluster, showed low adherence to the PHD recommendations. As mentioned, the resulting clusters mostly reflect the EAT-Lancet index’s ranking, except for Germany. This country clustered with the Netherlands and Sweden (brown cluster, as shown in the dendrogram on the left side of Figure 3), despite having the same score as Greece and Spain (21) and ranking higher than France (20).

##### 3.3.2.2 Regional patterns

In terms of macro-regions, the analysis showed a clear grouping of Southern European countries. On the contrary, the red cluster lacked a distinct geographical pattern, incorporating countries from both Eastern and Northern Europe. In addition, the brown cluster was not clearly defined, as indicated by the silhouette values. Germany (0.04) and Sweden (0.06) were placed between the brown and yellow cluster while the Netherlands (0.06) was between the brown and red clusters (Supplementary Table S7).

#### 3.3.3 Cluster analysis based on the WISH 2.0 score: gender differences

##### 3.3.3.1 The structure of clusters and their alignment with index ranking

**Figure 4:**
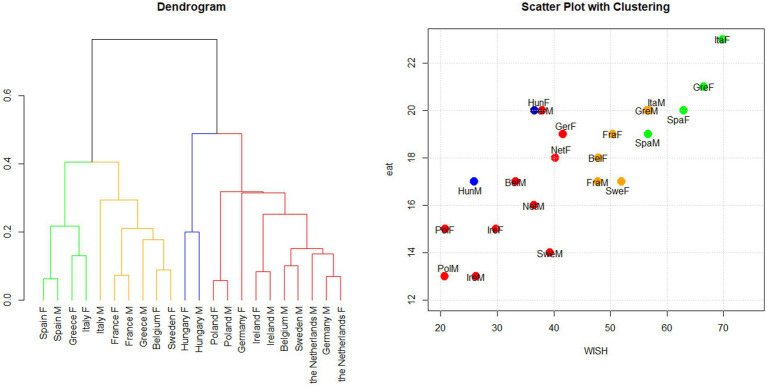
Hierarchical clustering based on the WISH 2.0 by gender. Cluster analysis was applied to the points assigned to each food category using the WISH 2.0 index system. Dendrogram and scatter plot showing the results of the cluster analysis conducted in the 11 PLAN’EAT countries by gender. The dendrogram shows how the countries are grouped according to clustering, while the scatter plot shows WISH 2.0 and EAT-Lancet indices’ scores for each country. The plots are colored according to the cluster groups. F: females; M: males.

Cluster analysis was conducted on the points assigned to each category of food consumed based on the WISH 2.0 system, stratified by gender. As shown in Figure 4, the results revealed four distinct clusters that mainly corresponded to the overall WISH 2.0 score rankings for each gender group (Supplementary Table S8).

The *green* cluster included the population groups with the highest aggregate scores and comprised Italian, Greek, and Spanish women and Spanish men. Men from Greece and Italy, men and women from France, women from Sweden and Belgium, and Greek and Italian men were placed in the *yellow* cluster, representing groups with intermediate scores. The *red* cluster consisted of groups with the lowest scores and included Swedish and Belgian men, with both gender groups from the Netherlands, Germany, Poland, and Ireland. The analysis also identified a distinct blue cluster of Hungarian men and women whose characteristics were different from those of the other countries (see dendrogram on the left of Figure 4).

##### 3.3.3.2 Gender differences

The *green* cluster mainly consisted of women from countries with the highest WISH 2.0 total scores, such as Italy, Greece, and Spain (Supplementary Table S8). The *yellow* cluster, on the other hand, included men from countries with the highest score (Greece and Italy) and women from countries with intermediate WISH 2.0 total scores (Sweden, France, Belgium). The *red* cluster primarily comprised men from countries with intermediate and low WISH 2.0 scores. These findings exemplified the generally higher scores achieved by women.

In greater detail, Spain, France, and Poland exhibited very similar scoring patterns for both genders, making them the first countries in which the two groups were combined into a cluster (green for Spain, yellow for France, and red for Poland, as shown in the dendrogram in Figure 4). A similar situation was observed for Ireland, Hungary, and the Netherlands, where men and women were grouped together. Even in the case of Germany, the two genders were grouped, but later in the analysis. The gender groups for other countries did not cluster together. Silhouette width values indicated that Italian men (0.11) were closer to the *green* cluster, which included Italian women. Similarly, Belgian women, with a silhouette value of 0.12, were also close to the *red* cluster, which included Belgian men. Finally, the silhouette value of Irish men (0.03) indicated that they were positioned almost exactly between the red and blue clusters (Supplementary Table S8).

##### 3.3.3.3 Regional patterns

The resulting clusters mostly reflected the division of European macro-regions. The *green* cluster represented women in the Southern European countries, while the *yellow* cluster incorporated different groups (men, women, or both) from different macro-regions (Southern, Northern, and Western). The *red* cluster included many Northern countries and some Western (Germany and the Netherlands) and Eastern countries.

#### 3.3.4 Cluster analysis based on the EAT-Lancet index score: gender differences

##### 3.3.4.1 The structure of clusters and their alignment with index ranking

**Figure 5:**
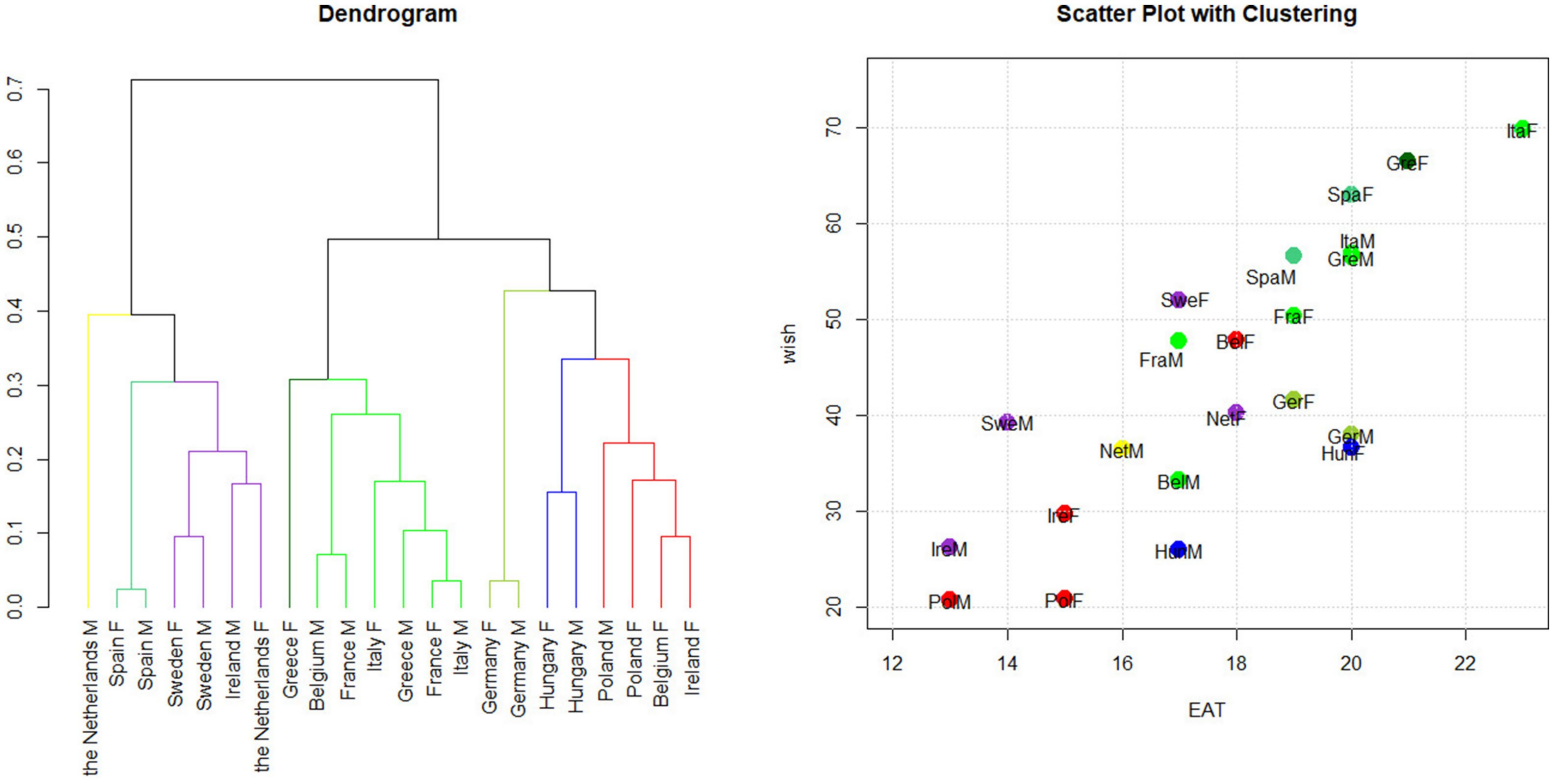
Hierarchical clustering based on the EAT-Lancet index by gender. Cluster analysis was applied to the points assigned to each food category using the EAT-Lancet index system. Dendrogram and scatter plot showing the results of the cluster analysis conducted in the 11 PLAN’EAT countries by gender. The dendrogram shows how the countries are grouped according to clustering, while the scatter plot shows EAT-Lancet and WISH 2.0 indices’ scores for each country. The plots are colored according to the cluster groups F: females; M: males.

Cluster analysis was performed on the points assigned to each food category according to the EAT-Lancet index, stratified by gender. As shown in Figure 5, eight clusters were identified, which did not align with the overall EAT-Lancet index ranking (Supplementary Table S9), resulting in a scattered pattern rather than clusters being combined. The *green* cluster predominantly consisted of groups with the highest EAT-Lancet total scores, such as Italian men and women and Greek men, but it also included groups with intermediate scores, such as French men and women and Belgian men. On the other hand, the *red* and *purple* clusters included the countries with the lowest scores such as men and women from Poland and Ireland and Swedish men. These clusters also included countries with intermediate scores such as Belgium, the Netherlands, and women from Sweden (Figure 5). Furthermore, the *blue* cluster (for Hungary), the *sea-green* cluster (for Spain), and the *olive-green* cluster (for Germany) comprised both gender groups from the same country.

##### 3.3.4.2 Gender differences

In most cases, men and women from the same country were placed in the same cluster. The strong similarity within the same country was highlighted by the early grouping that occurred, such as in the case of Spain and Germany. In other cases, such as for Italy and France, the grouping occurred later (Figure 5).

Nevertheless, the silhouette value for Italian women (0.04) indicated that this group was positioned between the *green* cluster, which included Italian men, and the cluster made of only Greek women. Furthermore, women from Belgium and the Netherlands seemed to be incorrectly placed as indicated by their negative silhouette widths (−0 13 and −0.02, respectively). In fact, Belgian women were placed closer to the *olive-green* cluster, which included both German gender groups, while Dutch women were closer to the *sea-green* cluster, including both Spanish men and women (Supplementary Table S9).

##### 3.3.4.3 Regional patterns

From a macro-regional perspective, Spain did not cluster with other Mediterranean countries (*green* cluster) in either the eight-group or three-group clustering. Surprisingly, Belgian men were part of the *green* cluster. Lastly, the *red* cluster comprised Northern and Eastern European countries, while the *purple* cluster comprised Northern and Western European countries. Three key unexpected findings emerged. Firstly, Hungary’s differences were less pronounced in this analysis than in the previous one. Secondly, Dutch men were markedly different from all other groups. Thirdly, Italian men and French women exhibited similar eating behaviors (see Figure 5).

#### 3.3.5 Cluster analysis based on food consumption

##### 3.3.5.1 The structure of clusters and their alignment with index ranking

**Figure 6:**
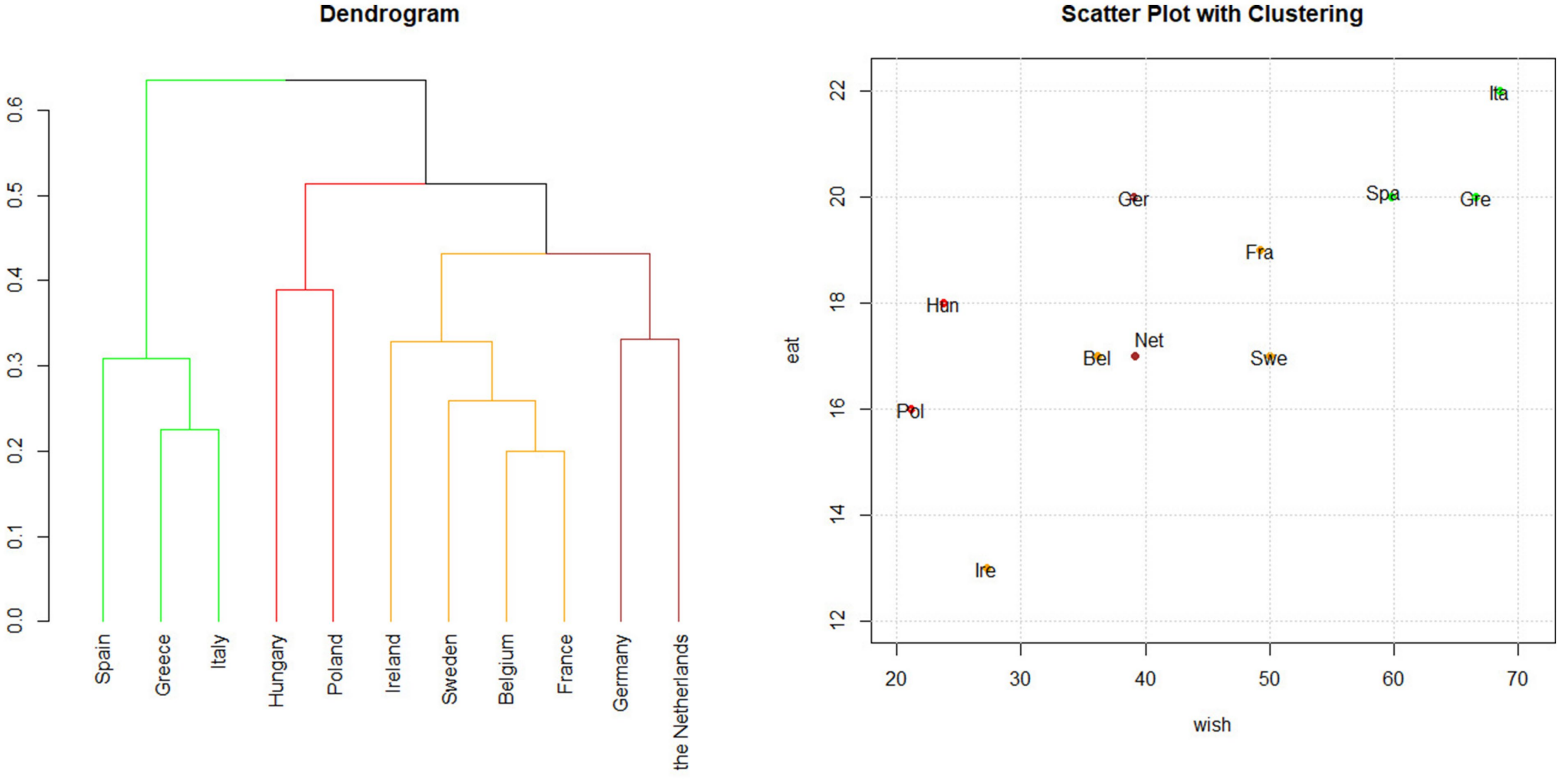
Hierarchical clustering based on food consumption. Cluster analysis was applied to the points assigned to each food category consumed. Dendrogram and scatter plot showing the results of the cluster analysis conducted in the 11 PLAN’EAT countries. The dendrogram shows how the countries are grouped according to clustering, while the scatter plot shows EAT-Lancet and WISH 2.0 indices’ scores for each country. The plots are colored according to the cluster groups.

Cluster analysis was conducted on the average daily consumption of each food category across the countries analyzed. The resulting clusters, presented in Figure 6, generally corresponded with the overall WISH 2.0 rankings (Supplementary Table S10).

The *green* cluster, comprising Italy, Greece, and Spain, achieved the highest scores in both indices. In contrast, the *red* cluster, consisting of Hungary and Poland, corresponded to the lowest WISH 2.0 scores. The *brown* cluster, consisting of Germany and the Netherlands, had intermediate WISH 2.0 score, whereas the EAT-Lancet index score was medium for the Netherlands and high for Germany. The *yellow* cluster, which included France, Sweden, Belgium, and Ireland, exhibited intermediate scores for both indices and low EAT-Lancet index score.

##### 3.3.5.2 Regional patterns

Cluster analysis of the average daily consumption of each food category revealed a well-defined regional grouping. Specifically, the *green* cluster consisted only of Southern European countries, the *yellow* cluster included Western and Northern European countries, the *brown* cluster comprised Western countries, and the *red* cluster included Eastern European countries. However, Germany and Hungary, with negative silhouette values (−0.06 and −0.01 respectively) were not well placed in their own clusters, being closer to the *yellow* one (Supplementary Table S10).

##### 3.3.5.3 Comparison with WISH 2.0 and EAT-Lancet index cluster analysis results

France was not grouped with the Mediterranean countries in the cluster analysis based on the EAT-Lancet index for the overall population and in the food consumption cluster analysis, whereas in the WISH 2.0 clustering, it was grouped with countries from the Mediterranean region. Germany and the Netherlands were consistently grouped together in the same cluster across the two analyzed indices, as well as in the cluster analysis based on average food category consumption. However, a distinctive pattern emerged for the EAT-Lancet index and the food category analysis, where these countries were grouped apart from the intermediate score cluster (*yellow*). Ireland was clustered with Poland in the EAT-Lancet index analysis for the overall population and with Hungary in the WISH 2.0 analysis. Meanwhile, Hungary and Poland were grouped together in the food consumption-based clustering. However, this grouping was characterized by very low silhouette values, indicating weak cohesion within the cluster (Supplementary Table S10).

## 4 Discussion

The purpose of this paper was to compare and analyze three dietary indices with different scoring systems: the original WISH, its updated version WISH 2.0 (both used to map dietary patterns in 11 European countries in the framework of the PLAN’EAT project), and the EAT-Lancet index. The findings of this study provided valuable insights into the differences between the indices (WISH/WISH 2.0 and EAT-Lancet). Furthermore, the work highlighted how each scoring system captures and evaluates dietary patterns in different ways.

Starting with their structural characteristics, WISH/WISH 2.0 and EAT-Lancet index differ in terms of the food categories included, the number of items, how foods are grouped, and the cut-offs values used to assign scores. Turning to the results, the overall low adherence to the PHD across the 11 countries analyzed indicates substantial room for improving alignment between current dietary habits and healthy, sustainable recommendations. This aspect should be strongly considered, given that dietary risks are among the leading causes of death and disability worldwide. Furthermore, diets that are low in plant-based foods and high in animal-based products (particularly red and processed meat) substantially contribute to environmental degradation (6). Therefore, the addition into the WISH 2.0 index of processed meat and alcoholic beverages, two categories for which the consumption is linked to chronic disease risk and environmental burden, demonstrated a stronger discriminatory power and better alignment with real food consumption data (24).

The cluster analysis applied to the scores obtained for each food category of the WISH 2.0 and EAT-Lancet index allowed to identify similarities and differences in the dietary patterns of the 11 European countries analyzed in this paper. However, both indices were based on cut-offs derived from reference ranges, which offer a simplified representation of food consumption. As stated by Wajiers et al. (38), this approach does not differentiate between intakes that fall significantly below or above the defined thresholds. This highlights the value of using food consumption data, which allows for precise quantification of intake and the identification of shared dietary patterns; accordingly, cluster analysis based on average food group intake was used as a reference to assess the performance of the indices.

Concerning the analysis, comparing the clusters to the ranking obtained by the indices, it was pointed out that country groups based on the WISH 2.0 index were more aligned with those derived from current food consumption data. This suggests that WISH 2.0 may better capture the diversity of dietary habits across countries, reinforcing its characteristic to be a reliable tool for assessing and mapping diet quality. One possible explanation for this is the difference in the scoring system. The EAT-Lancet index assigns a fixed score on a four-point scale for each food category, while the WISH uses a broader 10-point continuous scale. This wider scale may improve WISH’s ability to detect variation in dietary patterns.

In addition to this, it should be noted that even though the PHD was conceived to preserve human and planetary health, Beal et al. indicate that this dietary model does not adequately cover the recommended level of some micronutrients (e.g., vitamin B12, calcium, iron, and zinc), especially for vulnerable groups such as women of reproductive age (39). While WISH 2.0 was developed as a food group–based tool to assess dietary quality in terms of health and sustainability, it was not designed to capture micronutrient adequacy, which typically requires nutrient-level or biomarker-based assessment. Nonetheless, findings like those of Beal et al. (39) point to the complementary value of integrating micronutrient-focused analyses in future work, alongside food-based indices like WISH 2.0, to provide a more complete picture of diet quality.

Regarding the country-specific results, the higher overall scores for both the WISH 2.0 and the EAT-Lancet index in Southern European countries (Italy, Greece, and Spain) reinforce the connection between the Mediterranean dietary model and the principles outlined by the PHD recommendations (40). Among the remaining countries, several Western European nations formed a distinct cluster based on their WISH 2.0 scores. Meanwhile, the Eastern European countries (Hungary and Poland) were grouped together in the cluster analysis based on food consumption and were clustered with Ireland in the other analyses. All these three countries demonstrated the lowest levels of adherence to the PHD recommendations. Another noteworthy finding of this study was Hungary’s consistent classification as a standalone cluster in the gender-stratified analyses for both indices.

These findings suggest that, unlike the Southern European countries, no clearly defined clusters emerged for other European macro-regions, highlighting a greater heterogeneity in dietary patterns across these nations. These results confirm that Europe is a region characterized by a considerable dietary diversity, which has been shaped by different cultural, geographical, and historical influences (41). The regional differences and characteristics are not captured by the PHD, which provides a global framework for healthy eating (10). An adaptation of the PHD providing recommendations for European food consumption patterns is envisaged. This would ensure that this dietary model is both feasible and effective in the European context.

The variation in adherence to the PHD across European regions may be largely explained by underlying cultural and dietary traditions and food availability. As reported by Boujelbane et al., (42), in Northern and Eastern Europe, dietary patterns tend to emphasize food products such as red and processed meat, dairies, and refined grains, while consumption of plant-based foods such as fruits, vegetables, legumes, and nuts remains relatively low. These dietary aspects contributed to the overall lower adherence to the PHD observed in these areas, particularly among men. By contrast, Southern European countries such as Italy, Greece, and Spain demonstrated higher adherence, reflecting the enduring influence of the traditional Mediterranean diet. This diet encourages the consumption of plant-based foods, olive oil, and moderate fish intake, which are all core components of the PHD. These regional dietary norms, shaped by long-standing cultural practices, appear to play a key role in determining how closely people adhere to PHD recommendations.

Regarding the analysis of dietary habits among the two genders, it was highlighted that across all indices, women achieved higher scores than men, reflecting greater adherence to PHD recommendations.

This trend was further supported by the gender-stratified cluster analyses, which grouped together women with higher scores and, at the later time, the male groups of the same country. According to these findings, previous research consistently showed that women generally follow healthier dietary patterns and exhibit greater adherence to nutritional guidelines and health-conscious behaviors (43). Therefore, these findings suggest that public health interventions should be tailored not only to national dietary contexts but also to gender-specific dietary behaviors, especially in countries where intra-national gender gaps are more evident.

This study provides valuable insights to inform and complement existing EU and national dietary guidelines and sustainability strategies. The low overall adherence to the PHD observed across the 11 European countries highlights the discrepancy between current dietary behaviors and the principles promoted by European frameworks such as the Farm to Fork Strategy (8) and the EU Green Deal (7), which advocate healthier and more sustainable food systems. Furthermore, the regional clustering patterns, particularly the alignment of Mediterranean countries with higher adherence scores, suggest that PHD-aligned recommendations should be integrated into national guidelines that already emphasize plant-rich dietary traditions, such as the Mediterranean Diet. These results emphasize the importance of tailoring sustainability-oriented dietary policies to local contexts:—a direction increasingly reflected in evolving FBDGs across Europe.

This study has both strengths and limitations. Notably, it is the first study that evaluated dietary quality indices using official consumption data from the EFSA’s Comprehensive European Food Consumption Database (27) while simultaneously considering both health and sustainability dimensions. This is an important strength considering that EFSA remains the most comprehensive, standardized, and publicly accessible source of harmonized dietary data across EU countries (44). The EFSA database use allows for cross-country comparison, which is central to the aims of this study. However, it should be noted that food consumption data are currently being updated for some countries. Hence, the available data may not fully reflect the present dietary habits across all studied nations. This temporal variability is a known limitation. On the other hand, while some national dietary patterns may have changed since the last data collection, core consumption trends, particularly those shaped by cultural and structural factors, tend to be relatively stable over time. Future updates to the EFSA database or access to more recent national datasets would enhance the temporal relevance of dietary assessments and allow for validation or refinement of current findings. However, as the main aim of this study was to evaluate the performance of the selected dietary quality indices, the use of slightly outdated data is unlikely to have had a significant impact on the overall conclusions.

Another key contribution of this study is its broad European perspective, encompassing 11 countries from all four major European macro-regions. This wide geographical coverage enables a comprehensive analysis of dietary patterns across diverse cultural, social, and economic contexts. By including countries from Southern, Northern, Western, and Eastern Europe, the study offers valuable insights into regional differences in adherence to Planetary Health Diet recommendations and highlights the impact of local dietary habits and food environments. Future research should aim to include additional European countries and explore regional breakdowns and urban–rural differences to enrich the analysis. Additionally, even if not planned yet, efforts to validate the WISH 2.0 in non-European contexts, such as Asia, Africa, and other global regions, could be an added value to assess its generalizability and applicability across diverse dietary and cultural settings.

The most significant limitation of this study lies in the use of average consumption values derived from aggregated data. While the use of standardized pooled data from the EFSA Comprehensive Food Consumption Database ensures cross-country comparability, it decreases the ability to capture the full range of inter-individual variability in dietary patterns. This constraint is particularly relevant for food categories with narrow recommended intake ranges. Population-level aggregated data may cover meaningful differences within subgroups, as in the scores of processed meat and alcoholic beverages, which received zero points in all countries. In more depth, while the cut-offs in WISH 2.0 were developed based on robust health and sustainability evidence (24), their application to country-level mean values may hinder adherence among specific population subgroups. Such variation is evident for alcoholic beverages and processed meat, where their intake can greatly differ among individuals within the same population (45). This does not necessarily suggest that the cut-offs are too strict; rather, it reflects the insufficient granularity of average data, which can hide differences in adherence at the individual level. The use of individual-level raw data would have allowed for a more precise analysis of dietary habits and likely resulted in non-zero scores for certain categories, potentially influencing the overall index scores. This would lead to a more precise characterization of dietary behaviors and enable more nuanced assessments, including stratification by demographic or socio-economic variables. It would also facilitate the application of statistical analyses to assess significant differences and variability within and between population groups. Expanding data granularity and incorporating biomarker-based validation in future research could significantly enhance the reliability of intake estimates, particularly for food groups prone to underreporting or misclassification. The integration of metabolic risk indicators and specific biomarkers (46) alongside dietary indices would allow for a more robust assessment of the relationship between health outcomes and the sustainability principles of diets. Together, these methodological improvements would increase the interpretability and policy relevance of diet quality indices, supporting more targeted and evidence-based public health interventions.

Overall, the results of this study contributed to a more refined classification of dietary indices, offering a nuanced and holistic perspective on diet quality across the 11 European countries involved in the PLAN’EAT project. Future research should prioritize the use of individual-level consumption data and extend the analysis to a wider range of countries to enhance the robustness of index-based assessments, validate the findings of this study, and better inform regionally tailored interventions. In addition, given that WISH 2.0 is a newly developed index, future validation through sensitivity analyses to assess its predictive power and reliability would be recommended. Another area worth exploring is the potential inclusion of ultra-processed foods (UPFs) in WISH 2.0, given the growing evidence on the impact of UPFs on both public health and environmental sustainability (47). However, integrating UPFs into the WISH 2.0 framework would entail a significant methodological shift. Current food consumption datasets (e.g., EFSA Comprehensive European Food Consumption Database) are not structured around the NOVA classification system, and UPFs span multiple food categories with highly variable environmental impacts (48). Therefore, incorporating this dimension would require a comprehensive conceptual redesign of WISH 2.0, shifting the focus from food group adequacy to the degree of food processing.

## 5 Conclusion

The analysis of 11 European countries in this study revealed generally low adherence to the PHD, both in terms of promoting human health and protecting the environment. Clear geographical and gender differences were observed, with Southern European countries and women showing dietary patterns more closely aligned with recommended guidelines. Among the diet quality indices evaluated, WISH 2.0 emerged as a more accurate tool than the EAT-Lancet index, as it more effectively captures actual food consumption patterns. Furthermore, by including specific scoring for alcoholic beverages and processed meat consumption, WISH 2.0 offers an enhanced capacity to assess diet quality considering emerging public health concerns. This comparative approach revealed important differences in how dietary behaviors are assessed, the specific food categories emphasized, and the extent to which the indices align with health and sustainability goals. The analysis clarified the unique characteristics of each index while also highlighting their complementary potential in offering a more comprehensive evaluation of dietary patterns across various contexts. The use of cluster analysis enabled the grouping of countries based on shared dietary characteristics and index scores. This method provided a structured framework to uncover patterns and differences that may not have been evident when examining individual index scores alone. For example, the cluster analysis revealed regional and gender-specific dietary trends, offering insights into distinctive consumption patterns and their alignment with health and sustainability objectives as measured by the WISH 2.0 and EAT-Lancet index. By organizing countries into clusters with similar characteristics, the analysis offered a clearer understanding of how specific dietary behaviors contribute to overall diet quality and adherence to nutritional and PHD recommendations, as is the case for the Mediterranean countries of Southern Europe. Moreover, this approach made it possible to identify outliers and borderline cases, such as countries or sub-populations that did not fit neatly into pre-defined regional or dietary quality categories. This study highlights the value of diet quality indices, particularly WISH 2.0, in capturing country- and gender-specific adherence to the PHD across diverse European contexts. The findings offer actionable insights for policymakers, showing where alignment with health and sustainability recommendations is strongest and where targeted interventions are most needed. Given its enhanced discriminatory power and broader food category inclusion, WISH 2.0 may serve as a valuable tool for monitoring dietary transitions and informing evidence-based food policies. In particular, WISH 2.0 stands out as a practical and adaptable monitoring tool that can serve as a foundation for the development of targeted food policies and interventions. Integrating it into national and European monitoring systems could help track population-level dietary trends and evaluate progress toward public health and sustainability targets.

## Data Availability

Publicly available datasets were analyzed in this study. This data can be found here: the EFSA Comprehensive European Food Consumption Database: https://www.efsa.europa.eu/en/data-report/food-consumption-data; repository publicly available, no need for the accession numbers.
