# Supplementary material for: Mapping of the adherence to the planetary health diet in 11 European countries: comparison of different diet quality indices as a result of the PLAN’EAT project
Source: Front Nutr. 2025 Sep 17;12:1645824. doi: 10.3389/fnut.2025.1645824 (PMC12486606; doi:10.3389/fnut.2025.1645824)
Supplement: Supplementary file 1 [file Data_Sheet_1.zip › Supplementary Tables.pdf]

Supplementary Material

Table S1. EFSA food category inclusion to calculate the indexes.

**Food category for the indices**	**EFSA food categories**
Whole grains	Pasta whole meal<br>Gluten free bread, brown<br>Wheat bread and rolls, brown or wholemeal<br>Wheat bread and rolls, semi-brown<br>Wheat flour, brown<br>Rusk, wholemeal<br>Crisp bread, wheat, wholemeal<br>Wheat wholemeal flour<br>Biscuits, sweet, wheat wholemeal<br>Rye bread and rolls, wholemeal<br>Rye flour, wholemeal
Vegetables	Canned/jarred vegetables<br>Dried vegetables<br>Fermented or pickled vegetables<br>Processed tomato products<br>Vegetable puree or paste<br>Bulb vegetables<br>Flowering brassica<br>Flowers used as vegetables<br>Fruiting vegetables<br>Fungi, mosses, and lichens<br>Herbs and edible flowers<br>Leafy vegetables<br>Legumes with pod<br>Root and tuber vegetables<br>Sprouts, shoots and similar<br>Stems/stalks eaten as vegetables<br>Dried vegetables<br>Fermented or pickled vegetables<br>Vegetable puree or paste<br>Processed tomato products<br>Canned/jarred vegetables
Fruits	Fruit used as fruit<br>Dried apples<br>Dried apricots<br>Dried bananas<br>Dried dates<br>Dried figs<br>Dried mangoes<br>Dried pears<br>Dried prunes<br>Dried vine fruits (raisins etc.)<br>Mixed dried fruits
Dairy	Cheese<br>Fermented milk or cream

<table>
<tr><td></td><td colspan="3">Milk and dairy powders and concentrates
Milk, whey and cream</td></tr>
<tr><td>Beef and lamb</td><td rowspan="2">WISH Index
Mammals edible offal, non-muscle, other than liver and kidney
Mammals fat tissue
Mammals kidney
Mammals liver
Mammals other slaughtering products
Mammals meat
Animal meat dried,
Animal mechanically separated meat (MSM)
Canned-tinned meat
Marinated meat,
Meat specialties,
Preserved/processed fat tissues
Processed whole meat products
Sausages</td><td rowspan="2">WISH 2.0 Index
Mammals edible offal, non-muscle, other than liver and kidney
Mammals fat tissue
Mammals kidney
Mammals liver
Mammals other slaughtering products
Mammals meat</td><td>EAT Lancet Index
Bovine edible offal, non-muscle, other than liver and kidney
Bovine fat tissue
Bovine kidney
Bovine liver
Bovine other slaughtering products
Bovine fresh meat
Sheep edible offal, non-muscle, other than liver and kidney
Sheep fat tissue
Sheep kidney
Sheep liver
Sheep other slaughtering products
Sheep fresh meat
Bovine meat dried
Canned-tinned meat
Meat based spreadable-textured specialities
Cooked bovine
Raw bovine</td></tr>
<tr><td>Pork</td><td>EAT Lancet Index
Pig edible offal, non-muscle, other than liver and kidney
Pig fat tissue
Pig kidney
Pig liver
Pig other slaughtering products
Pig fresh meat
Pork meat dried
Pate pork liver
Preserved/processed fat tissues
Cooked pork
Raw pork
Sausages</td></tr>
<tr><td>Chicken</td><td colspan="3">Poultry edible offal, non-muscle, other than liver and kidney
Birds fat tissue
Poultry kidney
Poultry liver
Poultry other slaughtering products</td></tr>
</table>

	Birds meat
Eggs	Eggs and egg products
Fish	Fish (meat)<br>Fish and seafood processed<br>Fish offal<br>Molluscs
Legumes	Legumes<br>Canned or jarred legumes, Pulses flour<br>Nuts, oilseeds and oil fruits
Nuts	Dried nuts/seeds and related flours and powders<br>Nut/seeds paste/emulsion/mass
Unsaturated oils	Seed oils<br>Olive oils<br>Fish oil
Added sugar	Confectionery including chocolate<br>Sugar and other sweetening ingredients (excluding intensive sweeteners)<br>Water-based sweet desserts<br>Fruit and vegetable juices and nectars (including concentrates)<br>Breakfast cereals<br>Fine bakery wares<br>Beverages concentrates, Water based beverages<br>Spoonable desserts and ice creams (generic)
Saturated oils (only WISH and WISH 2.0)	Fats and oils from terrestrial animals<br>Dairy fats<br>Other plant oils<br>Fat emulsions and blended fats
Processed meat (only WISH 2.0)	Animal meat dried<br>Animal mechanically separated meat (MSM)<br>Canned-tinned meat<br>Marinated meat<br>Meat specialties<br>Preserved/processed fat tissues<br>Processed whole meat products<br>Sausages
Alcoholic beverages (only WISH 2.0)	Alcoholic beverages
Potatoes (only EAT)	Potatoes and similar

Table S2. The EAT Lancet index scoring system description

	**Target intake (reference interval)**	**3 points**	**2 points**	**1 point**	**0 points**	
**Emphasized intake**						
Whole grains	232	>232	116–232	58–116	<58	**3 points** = intake above target intake<br>**2 points** = lower limit of reference interval up to target intake<br>**1 point** = 50%–100% of lower limit of reference interval
Vegetables	300 (200–600)	>300	200–300	100–200	<100	

Fruits	200 (100–300)	>200	100–200	50–100	<50	**0 points** = <50% of lower limit of reference interval<br>*for legumes, nuts and fish (which have a lower limit of 0):<br>2 points: 100% - 50% of target intake<br>1 point: 50% - 25% of target intake<br>0 point: < 25% of the target intake
Legumes	75 (0–150)	>75	37.5–75	18.75–37.5	<18.75	
Nuts	50 (0–100)	>50	25–50	12.5–25	<12.5	
Fish	28 (0–100)	>28	14–28	7–14	<7	
Unsaturated oils	40 (20–80)	>40	20–40	10–20	<10	
**Limited intake**						
Dairy	250 (0–500)	<250	250–500	500–1000	>1000	**3 points**: intake below target intake<br>**2 points**: target intake to upper limit of reference interval<br>**1 point**: 100%–200% of upper limit of reference interval<br>**0 points**: >200% of upper limit of reference interval<br>*for added sugar:<br>2 points: 100% -200% of upper limit of reference interval<br>1 point: 200% - 400% of upper limit of reference interval<br>0 point: < 400% of upper limit of reference interval
Beef and lamb	7 (0–14)	<7	7–14	14–28	>28	
Pork	7 (0–14)	<7	7–14	14–28	>28	
Eggs	13 (0–25)	<13	13–25	25–50	>50	
Poultry	29 (0–58)	<29	29–58	58–116	>116	
Added sugar	31 (0–31)	<31	31–62	62–124	>124	
Potatoes	50 (0–100)	<50	50–100	100–200	>200	

Table S3. The WISH/WISH 2.0 index scoring system description. The two additional food categories processed meat and alcoholic beverages are reported in bold.

	**Recommended intake (g/day)**	**10 points**	**0 points**	**Ratio between 0 and 10**
Whole grains	125 (100–150)	≥125	<100	100-125
Vegetables	300 (200–600)	≥300	<200	200-300
Fruits	200 (100–300)	≥200	<100	100-200
Legumes	75 (0–100)	≥75	0	0-75
Unsaturated oils	40 (20–80)	≥40	<20 and >80	20-40
Dairy foods	250 (0–500)	250-500	>500	0-250
Nuts	50 (0–75)	50-75	>75	0-50
Eggs	13 (0–25)	≤13	>25	13-25
Chicken and other poultry	29 (0–58)	≤29	>58	29-58
Fish	28 (0–100)	28-100	>100	0-28
Red meat	14 (0–28)	≤14	>28	14-28
Added sugars	31 (0–31)	<31	>31	-
Saturated oils	11.8 (0–11.8)	<11.8	>11.8	
**Processed meat**	**2 – 4**	**<2**	**>4**	**2 - 4**
**Alcoholic beverages**	**0**	**0**	**>0**	**-**

Tables S4 – WISH and WISH 2.0 scores assigned to each country for each food category.

Country	Gender	Added sugar	Alcoholic beverages	Dairy foods	Eggs	Fish	Fruits	Legumes	Nuts	Poultry	Processed meat	Red meat	Saturated oils	Unsaturated oils	Vegetables	Whole grains	WISH WISH2.0
Belgium	Female	0.0	0.0	6.6	10.0	8.9	1.5	0.7	0.7	9.5	0.0	0.0	10.0	0.0	0.0	0.0	**48**
	Male	0.0	0.0	6.6	10.0	10.0	0.0	0.9	0.7	5.1	0.0	0.0	0.0	0.0	0.0	0.0	**33**
	Total	0.0	0.0	6.6	10.0	9.9	0.8	0.8	0.7	7.4	0.0	0.0	0.0	0.0	0.0	0.0	**36**
France	Female	0.0	0.0	9.3	7.1	10.0	1.8	0.8	0.6	7.4	0.0	0.0	10.0	0.0	3.5	0.0	**50**
	Male	0.0	0.0	9.2	6.1	10.0	2.1	1.5	0.8	4.3	0.0	0.0	10.0	0.0	3.7	0.0	**48**
	Total	0.0	0.0	9.3	6.7	10.0	1.9	1.1	0.7	6.1	0.0	0.0	10.0	0.0	3.6	0.0	**49**
Germany	Female	0.0	0.0	7.4	10.0	5.0	6.1	0.6	0.8	10.0	0.0	1.7	0.0	0.0	0.0	0.0	**42**
	Male	0.0	0.0	7.4	10.0	6.3	2.6	0.7	1.0	10.0	0.0	0.0	0.0	0.0	0.0	0.0	**38**
	Total	0.0	0.0	7.4	10.0	5.5	4.6	0.7	0.9	10.0	0.0	0.0	0.0	0.0	0.0	0.0	**39**
Greece	Female	10.0	0.0	7.9	10.0	8.9	4.9	0.6	1.2	9.1	0.0	0.0	10.0	3.9	0.0	0.0	**66**
	Male	0.0	0.0	8.4	7.5	10.0	0.0	1.2	0.9	10.0	0.0	0.0	10.0	7.9	0.5	0.0	**56**
	Total	10.0	0.0	8.1	9.3	10.0	1.5	0.9	1.1	9.8	0.0	0.0	10.0	5.9	0.0	0.0	**67**
Hungary	Female	0.0	0.0	8.8	4.6	4.1	4.0	1.6	1.2	0.9	0.0	0.0	10.0	0.0	1.4	0.0	**37**
	Male	0.0	0.0	9.6	0.0	4.2	1.9	1.7	1.3	0.0	0.0	0.0	0.0	1.6	5.7	0.0	**26**
	Total	0.0	0.0	9.2	1.0	4.1	3.0	1.7	1.2	0.0	0.0	0.0	0.0	0.0	3.5	0.0	**24**
Ireland	Female	0.0	0.0	9.1	8.2	8.1	0.0	1.3	0.6	2.5	0.0	0.0	0.0	0.0	0.0	0.0	**30**
	Male	0.0	0.0	10.0	5.1	8.7	0.0	1.9	0.6	0.0	0.0	0.0	0.0	0.0	0.0	0.0	**26**
	Total	0.0	0.0	10.0	6.6	8.4	0.0	1.6	0.6	0.0	0.0	0.0	0.0	0.0	0.0	0.0	**27**
Italy	Female	10.0	0.0	6.5	10.0	10.0	6.3	1.5	1.2	8.2	0.0	0.0	10.0	3.3	2.8	0.0	**70**
	Male	0.0	0.0	6.4	6.3	10.0	6.6	1.5	1.5	3.5	0.0	0.0	10.0	6.6	4.4	0.0	**57**
	Total	10.0	0.0	6.5	8.4	10.0	6.5	1.5	1.4	5.9	0.0	0.0	10.0	4.8	3.6	0.0	**69**
the Netherlands	Female	0.0	0.0	10.0	9.9	6.6	1.2	0.9	1.7	10.0	0.0	0.0	0.0	0.0	0.0	0.0	**40**
	Male	0.0	0.0	10.0	7.1	7.7	0.0	0.8	2.8	8.1	0.0	0.0	0.0	0.0	0.0	0.0	**36**
	Total	0.0	0.0	10.0	8.5	7.2	0.5	0.8	2.2	10.0	0.0	0.0	0.0	0.0	0.0	0.0	**39**
Poland	Female	0.0	0.0	5.1	0.0	4.5	0.0	0.9	0.3	10.0	0.0	0.0	0.0	0.0	0.0	0.0	**21**

	Male	0.0	0.0	3.5	0.0	6.5	0.0	1.2	0.2	9.4	0.0	0.0	0.0	0.0	0.0	0.0	**21**
	Total	0.0	0.0	4.3	0.0	5.5	0.0	1.1	0.2	10.0	0.0	0.0	0.0	0.0	0.0	0.0	**21**
Spain	Female	10.0	0.0	10.0	9.0	10.0	4.7	1.2	0.4	7.7	0.0	0.0	10.0	0.0	0.0	0.0	**63**
	Male	10.0	0.0	10.0	3.8	10.0	5.3	1.3	0.5	5.8	0.0	0.0	10.0	0.0	0.0	0.0	**57**
	Total	10.0	0.0	10.0	6.4	10.0	5.0	1.3	0.5	6.7	0.0	0.0	10.0	0.0	0.0	0.0	**60**
Sweden	Female	0.0	0.0	10.0	7.6	10.0	2.6	0.7	1.1	10.0	0.0	0.0	10.0	0.0	0.0	0.0	**52**
	Male	0.0	0.0	10.0	7.8	10.0	0.0	0.6	0.9	10.0	0.0	0.0	0.0	0.0	0.0	0.0	**39**
	Total	0.0	0.0	10.0	7.7	10.0	0.7	0.6	1.0	10.0	0.0	0.0	10.0	0.0	0.0	0.0	**50**

Table S5 – EAT-Lancet index scores assigned to each country for each food category.

**Country**	**Gender**	**Added sugar**	**Beef and lamb**	**Dairy foods**	**Eggs**	**Fish**	**Fruits**	**Legumes**	**Nuts**	**Pork**	**Potatoes**	**Poultry**	**Unsaturated oils**	**Vegetables**	**Whole grains**	**EAT**
Belgium	Female	2	1	3	3	2	2	0	0	0	2	2	0	1	0	**18**
	Male	2	0	3	3	3	2	0	0	0	1	2	0	1	0	**17**
	Total	2	0	3	3	2	2	0	0	0	2	2	0	1	0	**17**
France	Female	2	0	3	2	3	2	0	0	0	2	2	1	2	0	**19**
	Male	2	0	3	2	3	2	0	0	0	1	2	0	2	0	**17**
	Total	2	0	3	2	3	2	0	0	0	2	2	1	2	0	**19**
Germany	Female	2	2	3	3	1	2	0	0	0	3	3	0	0	0	**19**
	Male	2	2	3	3	2	2	0	0	0	3	3	0	0	0	**20**
	Total	2	2	3	3	2	2	0	0	0	3	3	0	0	0	**20**
Greece	Female	3	0	3	3	2	2	0	0	1	2	2	2	1	0	**21**
	Male	2	0	3	2	3	1	0	0	0	2	3	2	2	0	**20**
	Total	3	0	3	2	3	2	0	0	0	2	2	2	1	0	**20**
Hungary	Female	2	3	3	2	1	2	0	0	0	2	2	1	2	0	**20**
	Male	2	1	3	1	1	2	0	0	0	2	1	2	2	0	**17**
	Total	2	2	3	2	1	2	0	0	0	2	1	1	2	0	**18**
Ireland	Female	2	0	3	2	2	1	0	0	0	2	2	0	1	0	**15**

	Male	2	0	2	2	2	1	0	0	0	2	1	0	1	0	**13**
	Total	2	0	2	2	2	1	0	0	0	2	1	0	1	0	**13**
Italy	Female	3	0	3	3	3	2	0	0	0	3	2	2	2	0	**23**
	Male	2	0	3	2	3	2	0	0	0	2	2	2	2	0	**20**
	Total	3	0	3	2	3	2	0	0	0	3	2	2	2	0	**22**
the Netherlands	Female	2	1	2	2	2	2	0	0	0	3	3	0	1	0	**18**
	Male	1	0	2	2	2	1	0	1	0	3	2	0	1	1	**16**
	Total	2	0	2	2	2	2	0	0	0	3	2	0	1	1	**17**
Poland	Female	2	1	3	1	1	1	0	0	0	2	3	0	1	0	**15**
	Male	2	0	3	1	2	0	0	0	0	1	2	1	1	0	**13**
	Total	2	0	3	1	2	1	0	0	0	2	3	1	1	0	**16**
Spain	Female	3	1	2	2	3	2	0	0	0	3	2	1	1	0	**20**
	Male	3	0	2	2	3	2	0	0	0	3	2	1	1	0	**19**
	Total	3	1	2	2	3	2	0	0	0	3	2	1	1	0	**20**
Sweden	Female	2	1	2	2	3	2	0	0	0	2	3	0	0	0	**17**
	Male	2	0	2	2	3	1	0	0	0	1	3	0	0	0	**14**
	Total	2	1	2	2	3	2	0	0	0	2	3	0	0	0	**17**

Table S6 – The 11 PLAN'EAT countries sorted by WISH 2.0 with the membership cluster and silhouette values from the cluster analysis applied to the points assigned to each category of food consumed according to the WISH 2.0 index system.

Country	WISH 2.0	cluster	neighbour	silhouette widths
Italy	68.61	green	red	0.45
Greece	66.58	green	yellow	0.20
Spain	59.80	green	yellow	0.30
Sweden	50.00	yellow	green	0.14
France	49.29	green	red	**0.07**
the Netherlands	39.22	yellow	red	0.35
Germany	39.05	yellow	red	0.42
Belgium	36.14	yellow	red	0.43
Ireland	27.23	red	yellow	**0.08**
Hungary	23.72	red	yellow	0.39
Poland	21.09	yellow	red	0.19
MEAN	43.70			

Table S7 – The 11 PLAN'EAT countries sorted by EAT-Lancet with the membership cluster and silhouette values from the cluster analysis applied to the points assigned to each category of food consumed according to the EAT-Lancet index system.

Country	EAT-Lancet	cluster	neighbour	silhouette widths
Italy	22	green	yellow	0.50
Greece	20	green	yellow	0.29
Spain	20	green	brown	0.31
Germany	20	brown	yellow	**0.04**
France	19	yellow	green	0.21
Hungary	18	yellow	red	0.33
Belgium	17	yellow	red	**0.08**
the Netherlands	17	brown	red	**0.06**
Sweden	17	brown	yellow	**0.06**
Poland	16	red	yellow	0.14
Ireland	13	red	yellow	0.22
MEAN	18.09			

Table S8 – The 11 PLAN'EAT countries by gender sorted by WISH 2.0 with the membership cluster and silhouette values from the cluster analysis applied to the points assigned to each category of food consumed according to the WISH 2.0 index system.

Country	Gender	WISH 2.0	cluster	neighbour	silhouette widths
Italy	Female	69.83	green	yellow	0.34
Greece	Female	66.49	green	yellow	0.37
Spain	Female	62.97	green	yellow	0.45
Italy	Male	56.87	yellow	green	**0.11**
Spain	Male	56.68	green	yellow	0.37
Greece	Male	56.49	yellow	green	0.28
Sweden	Female	51.97	yellow	green	0.24
France	Female	50.42	yellow	green	0.37
Belgium	Female	47.85	yellow	red	**0.12**
France	Male	47.74	yellow	blu-hungary	0.28
Germany	Female	41.57	red	yellow	0.28
the Netherlands	Female	40.30	red	yellow	0.39
Sweden	Male	39.28	red	yellow	0.28
Germany	Male	37.99	red	yellow	0.38
Hungary	Female	36.58	blu-hungary	yellow	0.18
the Netherlands	Male	36.47	red	yellow	0.35
Belgium	Male	33.23	red	yellow	0.27
Ireland	Female	29.75	red	yellow	0.31
Ireland	Male	26.26	red	blu-hungary	**0.03**
Hungary	Male	25.96	blu-hungary	red	0.36
Poland	Female	20.83	red	blu-hungary	0.35
Poland	Male	20.72	red	blu-hungary	0.38
MEAN	Male	39.79			
MEAN	Female	47.14			
MEAN	ALL	43.47			

Table S9 – The 11 PLAN'EAT countries by gender sorted by EAT-Lancet with the membership cluster and silhouette values from the cluster analysis applied to the points assigned to each category of food consumed according to the EAT-Lancet index system.

Country	Gender	EAT-Lancet	cluster	neighbour	silhouette widths
Italy	Female	23	green	singleton GF	0.04
Greece	Female	21	singleton GF	green	0.00
Italy	Male	20	green	blu-hungary	0.42
Greece	Male	20	green	red	0.33
Spain	Female	20	seagreen-spain	purple	0.89
Germany	Male	20	olivegreen-germany	red	0.85
Hungary	Female	20	blu-hungary	green	0.32
France	Female	19	green	blu-hungary	0.42
Spain	Male	19	seagreen-spain	purple	0.89
Germany	Female	19	olivegreen-germany	red	0.86
Belgium	Female	18	red	olivegreen-germany	**-0.13**
the Netherlands	Female	18	purple	seagreen-spain	**-0.02**
Belgium	Male	17	green	red	**0.06**
France	Male	17	green	red	0.36
Hungary	Male	17	blu-hungary	green	0.38
Sweden	Female	17	purple	seagreen-spain	0.29
the Netherlands	Male	16	singleton NM	purple	0.00
Ireland	Female	15	red	green	0.31
Poland	Female	15	red	olivegreen-germany	0.23
Sweden	Male	14	purple	seagreen-spain	0.39
Ireland	Male	13	purple	red	**0.08**
Poland	Male	13	red	green	0.22
MEAN	Male	16.91			
MEAN	Female	18.64			
MEAN	ALL	17.77			

Table – S10 the 11 PLAN'EAT countries sorted by WISH 2.0 and by EAT-Lancet with the membership cluster and silhouette values from the cluster analysis applied to the food consumption values.

countries sorted by WISH 2.0					
Country	WISH 2.0	EAT	cluster	neighbour	silhouette widths
Italy	68.61	22	green	yellow	0.38
Greece	66.58	20	green	yellow	0.20
Spain	59.80	20	green	yellow	0.22
Sweden	50.00	17	yellow	brown	**0.09**
France	49.29	19	yellow	green	0.13
the Netherlands	39.22	17	brown	yellow	0.13
Germany	39.05	20	brown	yellow	**-0.06**
Belgium	36.14	17	yellow	brown	0.17
Ireland	27.23	13	yellow	brown	0.25
Hungary	23.72	18	red	yellow	**-0.01**
Poland	21.09	16	red	yellow	**0.01**
MEAN	43.70	18.09			

countries sorted by EAT-Lancet					
Country	EAT	WISH 2.0	cluster	neighbor	silhouette widths
Italy	22	68.61	green	yellow	0.38
Greece	20	66.58	green	yellow	0.20
Spain	20	59.80	green	yellow	0.22
Germany	20	39.05	brown	yellow	0.13
France	19	49.29	yellow	green	0.13
Hungary	18	23.72	red	yellow	**-0.01**
Sweden	17	50.00	yellow	brown	**0.09**
the Netherlands	17	39.22	brown	yellow	**-0.06**
Belgium	17	36.14	yellow	brown	0.17
Poland	16	21.09	red	yellow	**0.01**
Ireland	13	27.23	yellow	brown	0.25
MEAN	18.09	43.70			
